# Design of the ZWOT-CASE study: an observational study on the effectiveness of an integrated programme for cardiovascular risk management compared to usual care in general practice

**DOI:** 10.1186/s12875-019-1039-z

**Published:** 2019-11-01

**Authors:** Suzanne Marchal, Monika Hollander, Marieke Schoenmakers, Michiel Schouwink, Jorik R. Timmer, Henk J. G. Bilo, Olof Schwantje, Arnoud W. J. van ’t Hof, Arno W. Hoes

**Affiliations:** 1 Julius Center for Health Sciences and Primary Care/ University Medical Center Utrecht and Utrecht University, Huispost Str. 6.131, PO Box 85500, 3508 GA Utrecht, The Netherlands; 2 Medrie, Dr. Klinkertweg 18, PO Box 40099, 8004 DB Zwolle, The Netherlands; 3 Isala, Dokter van Heesweg 2, PO Box 10400, 8000 GK Zwolle, The Netherlands; 4 Center for Integrated Care, Dr. Spanjaardweg, 118025 BT Zwolle, The Netherlands; 5 General Practitioners Region Region of Zwolle, Zwolle, The Netherlands; 6 Department of Cardiology, Maastricht University Medical Center, Maastricht, The Netherlands; 7 Zuyderland Medical Center, department of Cardiology, Heerlen, The Netherlands

**Keywords:** Cardiovascular disease, Prevention, Primary care, Integrated care/ disease management programme

## Abstract

**Background:**

Cardiovascular diseases (CVD) contribute considerably to mortality and morbidity. Prevention of CVD by lifestyle change and medication is important and needs full attention.

In the Netherlands an integrated programme for cardiovascular risk management (CVRM), based on the Chronic Care Model (CCM), has been introduced in primary care in many regions in recent years, but its effects are unknown.

In the ZWOT-CASE study we will assess the effect of integrated care for CVRM in the region of Zwolle on two major cardiovascular risk factors: systolic blood pressure (SBP) and low-density lipoprotein cholesterol (LDL-cholesterol) in patients with or at high risk of CVD.

**Methods:**

This study is a pragmatic observational study comparing integrated care for CVRM with usual care among patients aged 40–80 years with CVD (*n* = 370) or with a high CVD risk (n = 370) within 26 general practices. After 1 yr follow-up, primary outcomes (SBP and LDL-cholesterol level) are measured. Secondary outcomes include lifestyle habits (smoking, dietary habits, alcohol use, physical activity), risk factor awareness, 10-year risk of cardiovascular morbidity or mortality, health care consumption, patient satisfaction and quality of life.

**Conclusion:**

The ZWOT-CASE study will provide insight in the effects of integrated care for CVRM in general practice in patients with CVD or at high CVD risk.

**Trial registration:**

The ZWOlle Transmural Integrated Care for CArdiovaScular Risk Management Study; ClinicalTrials.gov; Identifier: NCT03428061; date of registration: 09-02-2018; This study has been retrospectively registered.

## Background

Cardiovascular disease (CVD) is still the leading cause of death in the world and the second cause of death in Western societies [1, 2]. Because of the ageing population, the prevalence and associated costs of CVD are expected to increase considerably [3]. Moreover, due to adverse lifestyle factors an increase in the prevalence of obesity and diabetes mellitus has been observed in the past 25 years. In addition, we are dealing with high levels of persistent smoking. These trends partly negate the beneficial effect of improvements in blood pressure and lipid control achieved in the last decades [4]. Therefore, prevention of (re)occurrence of CVD remains crucial.

To prevent CVD, national and international guidelines for cardiovascular risk management (CVRM) provide clinical and organisational recommendations [5–7]. However, implementation of evidence-based guidelines is far from optimal and treatment goals are often not achieved [8–10]. Thus, despite the availability of accurate guidelines, CVRM needs improvement.

In the Netherlands, integrated care programmes were introduced to implement CVRM in general practices in recent years. The integrated CVRM care programme includes a patient-centered focus, use of clinical information systems, execution by practice nurses (PNs) and systematic invitation of patients for a CV risk assessment. The integrated CVRM care programme is based on the Chronic Care Model (CCM) that focuses on informed and activated patients who interact with trained, proactive practice teams [11, 12].

Integrated programmes compose a promising procedure to enhance chronic care and management of cardiovascular risk factors. However, solid evidence on the effect of integrated care programmes for CVRM in primary care on outcomes such as blood pressure, cholesterol levels, smoking status and cardiovascular risk is limited [13–15].

A nurse-coordinated CVD prevention programme (Euro Action) has been shown to improve blood pressure targets [16]. Also, a disease management programme for patients with coronary heart disease (CHD) in primary care led to more adequate treatment of blood pressure and cholesterol compared to usual care and to a better controlled hypertension in high risk patients [17]. Additionally, a tailored implementation of cardiovascular risk management in general practice increases physical activity in cardiovascular patients, but did not affect other cardiovascular risk factors [18]. More recently, a multicomponent cardiovascular prevention programme did not improve the overall risk profile in older adults free from CVD in primary care, compared to usual care [19]. However, the usefulness of these previous studies is restricted by the heterogeneity in study designs, variety in the interventions tested and in the target populations. Most of the previous studies evaluated only a limited number of elements of the disease management programme for CVRM, such as lifestyle treatment or educational interventions. Rarely, effects of an integrated approach programme has been evaluated by analysing clinical parameters before and after implementation of the intervention, but adequate comparisons with control groups are lacking [20].

In the ZWOT-CASE study (ZWOlle inTegrated care for CArdiovaScular risk managEment study) we will investigate the effect of integrated care for CVRM compared to usual care within general practices in the region of Zwolle in the eastern part of the Netherlands. In this paper we describe the design of the study.

## Methods/ design

### Study aim

The primary aim of the ZWOT-CASE study is to investigate whether the execution of an integrated primary care programme for CVRM in general practice leads to a more favourable CV risk profile in patients with known CVD or at high CVD risk as compared to usual care.

### Study design

The ZWOT-CASE study is a prospective pragmatic observational study, performed among 740 patients with known CVD or at high CVD risk in general practice, comparing integrated care for CVRM with usual care. Patients in the usual care group are matched with patients in the intervention group according to age, gender and risk group (high CV risk or CVD). After 1 yr of follow-up outcomes are compared between the intervention group and the usual care group. Primary outcomes are levels of systolic blood pressure and LDL-cholesterol. The study was reviewed by the Isala hospital Review Board and exempted from full assessment under the Medical Research Involving Human Subjects Act on the 16th of June 2016.

### Setting

The study is performed in the Zwolle region in the Netherlands. This region includes 56 general practices (solo, duo and group practices) with in total 157 general practitioners (GPs), which are all affiliated to a care group 'Medrie’. Integrated care for CVRM is implemented in this region and coordinated by this care group by providing a practical guideline for the implementation of integrated care for CVRM, offering training to the PNs and organizing yearly benchmark meetings. Furthermore, all general practices collaborate with the same regional hospital (Isala Hospital) with dedicated medical specialists involved in organizing integrated care for CVRM. The care group reached an agreement on integrated care for CVRM with the regionally largest health care insurance company for 3 yrs. Implementation of other disease management programmes e.g. for diabetes mellitus and COPD is also organized by the same care group.

From January 2016, all practices were given the opportunity to participate in the integrated care for CVRM. Every 3 months there was a possibility to start with the programme. Participation was not mandatory. Consequently, the intervention was not randomly allocated to the practices. Prior to our study, approximately two third of the general practices (*n* = 37) chose to implement integrated care for CVRM, while the remaining general practices (*n* = 19) will continue usual care due to a variety of reasons. The practices in the usual care group will not have the opportunity to start with the integrated care for CVRM during the study.

This allowed the opportunity to compare integrated care for CVRM (intervention group) with usual care.

### The integrated CVRM programme

The intervention under study is the integrated care programme for CVRM (see Table 1), based on the Dutch CVRM guideline and the practical manual for CVRM provided by the Dutch Society of General Practitioners [7, 21]. The intervention includes features of the Chronic Care Model (CCM), such as self-management support (help patients to set limited goals and identify barriers to reach their goals), regular follow-up, registration of patient data in clinical information systems, a structured, nurse-led health care organization and easy accessible consultation of a specialist [22]. The aim of the integrated care programme for CVRM is to decrease the risk of CVD for patients with a high CV risk or history of CVD by lifestyle treatment and medication if needed. Treatment goals are according to the Dutch guideline for CVRM, including systolic blood pressure < 140/90 mmHg, LDL-cholesterol < 2,5 mmol/L, no smoking, BMI ≤ 25 kg/m2 (< 70 years) or ≤ 30 kg/m2 (≥ 70 years), > 5 days a week moderate intense physical activity > 30 min/day, and a healthy diet (daily 150–200 g vegetables and 200 g fruit; daily 30–40 g dietary fibers; twice a week 100–150 g fish, at least once fatty fish; maximum of 6 g salt per day; maximum of 2 (men) or 1 (women) alcohol consumptions per day).

**Table 1:** Elements of the integrated care for CVRM

Element	Contents
Systematic selection of target population	Systematic screening of practice population based on ICPC-codes
	Systematic screening of practice population based on ATC-codes
	Check of medical records according to in- and exclusion criteria of the programme
Active invitation of patients for the programme	Active invitation for an intake consultation by letter
	Reminder in case of no response
Collaboration with different disciplines	Well trained practice nurses, supervised by GPs
	Optional involvement of physiotherapist or dietician
	Online consultation of medical specialist
Data registration in multidisciplinary information system for integrated care (KIS, Portavita®)	Including data on laboratory measurement, intake consultation and follow-up controls
Benchmark meetings	Comparison op patient data of general practice with national data
Laboratory measurement (prior to intake consultation)	Lipids (total cholesterol, HDL-cholesterol, TC/HDL-cholesterol ratio, LDL-cholesterol, triglycerides)
	Renal function (creatinine, GFR estimated by MDRD)
	Glucose
Intake consultation	
Interview	Cardiovascular complaints
	Family history of CVD
	Medication adherence
	Lifestyle
	Motivation to change behaviour
Physical examination	Length, weight, BMI and waist circumference
	Blood pressure
	Pulse rate
Estimation of 10-years cardiovascular risk	Based on the risk chart in the Dutch guideline
cIndividual treatment goals	By shared decision making
General lifestyle advice	According to physical activity and diet
Medication (initiated or adapted if necessary)	Blood pressure lowering drugs
	Lipid lowering drugs
	Anticoagulants
Referral (if necessary)	Smoking cessation programmes
	Dietician
	Exercise programmes
	Physiotherapist
	Medical specialist
Regular follow-up	Evaluation of personal goals
	Adjustment of treatment

#### Organisation strategies of the integrated CVRM programme

The organisation strategies of integrated CVRM programme include:

- Systematic identification of patients eligible for CVRM
- Active invitation of patients for the programme
- Regular follow-up of patients
- Collaboration with different disciplines in the health care chain
- Registration of data in an information system for integrated care

General practices need to systematically organize their practice and identify the eligible population prior to implementation of integrated care for CVRM, based on a regional protocol (‘Organized Practice’) provided by the care group. According to this protocol patients with a history of CVD, at high CV risk (> 10%) or prescribed blood pressure or lipid lowering drugs are included in the programme. To identify the patients, the practice population is systematically screened based on International Classification of Primary Care (ICPC)-coded diagnoses and on Anatomical Therapeutic Chemical-codes (ATC-codes), a classification system for drugs (Tables 2, 3 and 4). Since they already are included in a separate disease management programme, patients with diabetes mellitus (DM) with ICPC-codes T90.01 (DM type I) and T90.02 (DM type II) are not included in the CVRM care programme. Subsequently, medical records of identified patients are manually checked to define whether they meet the in- and exclusion criteria.

**Table 2:** ICPC-coded diagnoses for patients with cardiovascular disease

Diagnosis	ICPC-code
Angina pectoris	K74/ K74.01/ K74.02
Acute myocardial infarction	K75
Other/chronical ischemic heart disease	K76
Coronary sclerosis	K76.01
Previous myocardial infarction (> 4 weeks ago)	K76.02
Transient ischaemic attack (TIA)	K89.01
Cerebral infarction	K90.3
Intermittent claudication	K92.01
Aneurysm aortae	K99.01

**Table 3:** ICPC-coded diagnoses for patients with high (> 10%) cardiovascular risk

Diagnosis	ICPC-code
Hypertension without organ damage	K86.00
Hypertension with organ damage	K87.00
Disorder of lipid metabolism	T93.00
Hypercholesterolemia	T93.01
Mixed hyperlipidaemia	T93.03
Familial hypercholesterolemia/−lipidaemia	T93.04
Rheumatoid arthritis	L88.01
M. Bechterew	L88.02
Psoriatic arthritis	S91.00

**Table 4:** ATC-codes

Medicine	ATC-code
Antithrombotic agents	B01
Cardiac therapy	C01
Blood pressure lowering drugs	C02
Diuretics	C03
Beta blocking agents	C07
Calcium channel blockers	C08
Agents acting on the renin-angiotensin system	C09
Lipid modifying agents	C10A

Once the eligible patients are identified, patients are actively invited by mail for an intake consultation. In this letter, patients are informed about the CVRM programme and invited to make an appointment for an intake consultation. If a patient does not respond to the invitation letter, a reminder is sent.

After the intake consultation, patients are monitored on a regular base in general practice. The frequency of follow-up visits depends on cardiovascular risk and treatment goals of individual patients, but a follow-up visit should be performed at least once a year.

Collaboration with several disciplines in the health care chain, such as GPs, medical specialists, practice nurses and -assistants, dieticians and physiotherapists, is an important focus of the CVRM programme. General practices implementing the integrated care intervention have well trained PNs, who identify the patients, review medical records and interview and examine the patients. All the PNs followed basic training including basic education in CVRM. In addition, some of the PNs followed a specialization course in CVRM, but this is not obligatory. The GP supervises the PNs. A dietician or physiotherapist may be involved if necessary. Also, a hospital specialist can be consulted easily online if necessary. If other disciplines are involved, they are given access to the patient data collected in a multidisciplinary information system for integrated care (KIS, Portavita®). This KIS is also used as a communication platform between the disciplines.

All patient data collected during the intake visit and follow-up visits will be registered in the KIS. These data will be used for benchmark purposes, including comparison of mean systolic blood pressure and LDL-cholesterol levels and smoking rates per practice with national data. Practices receive a benchmark report once a year and benchmark meetings will be organized by the care group.

#### Integrated CVRM programme for individual patients

For individual patients, the integrated CVRM programme includes:

- An intake consultation
- Regular follow-up visits
- Options for referral to get support in changing lifestyle

All eligible patients are invited for an individual face-to-face intake consultation at the general practice. Prior to the consultation, lipids (total cholesterol, HDL-cholesterol, TC/HDL-cholesterol ratio, LDL-cholesterol, triglycerides), renal function (creatinine, glomular filtration rate (GFR) estimated by the formula based on the Modification of Diet in Renal Disease study (MDRD)) and glucose are measured. The consultation consists of several components, including an interview to assess cardiovascular complaints, family history of CVD and difficulties with taking medication. Further, smoking habits, diet, alcohol, physical activity and psychological stress are assessed, as well as the patient’s motivation to change any factor if needed (Table 5).

**Table 5:** Assessment of lifestyle during intake consultation

Assessment of lifestyle	
Smoking	• Units per day
	• Smoking history
	• Attempts to quit
	• Motivation to quit
Dietary habits	• Knowledge of healthy dietary habits
	• Insight into own dietary habits
	• Necessity to change dietary habits
	• Motivation to change dietary habits
Alcohol use	• Units per week
	• Knowledge of effects of alcohol use
	• Insight into own alcohol use
	• Necessity to change alcohol use
	• Motivated to change alcohol use
Physical activity	• Days a week
	• Knowledge of importance of physical activity
	• Insight into own physical activity
	• Necessity to change physical activity
	• Motivated to change physical activity
Stress	• Stress symptoms > 3 months
	• Insight into own stress

Physical examination includes measurement of length, weight, (calculation of) BMI, waist circumference, blood pressure (manual or electronic oscillometric measurement, at least 2 measurements with an interval of 1–2 min) and pulse rate.

For patients without CVD an up-to-date 10-years CV morbidity and mortality risk based on the risk chart in the Dutch guideline (based on the SCORE risk function) will be estimated [5].

During the intake visit, individual treatment goals are determined, regarding smoking, physical activity, dietary habits, weight, BMI, blood pressure and LDL-cholesterol. These treatment goals are set by shared decision making between the caregiver and the patient based on the Dutch guideline for CVRM within the context of a patient’s personal preferences. If indicated, treatment with medication, including blood pressure and lipid lowering drugs, and anticoagulants will be initiated. All patients will be given general lifestyle advice by the PN. Patients not achieving a healthy lifestyle according to the Dutch guideline can be referred to smoking cessation programmes, dieticians and exercise programmes or a physiotherapist to get support in changing their lifestyle.

After the intake consultation, patients will be monitored on a regular base in general practice to evaluate and when necessary adjust their personal goals. At least once yearly, all measurements including estimation of the 10-years CV risk will be repeated.

### Usual care

Usual care is based on the Dutch CVRM guideline, describing how to calculate the CV risk and advice to lower this risk by lifestyle intervention and/or medication. However systematic identification of patients eligible for CVRM, actively inviting patients for a visit, regular follow-up and standardized collaboration with other disciplines in the health care chain are not routinely part of usual care. Usual care practices may work with a PN. Most PNs in the Netherlands have had a basic training in CVRM. Furthermore, data are not registered in an information system for integrated care and GPs do not participate in benchmark meetings.

### ZWOT-CASE study population

The ZWOT-CASE study population will consist of a subgroup of 370 patients from the integrated CVRM care group (intervention) and 370 patients in the usual care group. Both groups consist of respectively i) 185 patients with known CVD and ii) 185 patients with a high (> 10%) 10 yr risk of CVD morbidity and mortality based on the Dutch Guideline for CVRM [7, 23, 24].

Inclusion criteria for patients with CVD:

- Patients with a history of atherosclerotic CVD defined as documented angina pectoris, myocardial infarction, chronic ischemic heart disease, coronary sclerosis, transient ischaemic attack (TIA), cerebral infarction, intermittent claudication or aneurysm of the abdominal aorta
- The CV risk of the patient is managed in primary care, not in the hospital or outpatient clinic by a medical specialist
- Age between 40 and 80 years

Inclusion criteria for high risk patients:

- Use of blood pressure lowering or lipid lowering drugs
- A 10 -years CV risk > 10%, based on the Dutch guideline for CVRM and i) either 1 strongly cardiovascular risk enhancing factor or 2 mildly cardiovascular risk enhancing factors (see Table 6) or ii) > 1 CV risk factor (current smoking, SBP > 140 mmHg, LDL >  2.5 mmol/L, TC/HDL-ratio > 8, chronic renal impairment (age <  65 years: eGFR < 60 ml/min/1,73 m2; age ≥ 65 years: eGFR < 45 ml/min/1,73 m2, and/or (micro)albuminuria).
- A 10-year CV risk of > 20% and > 1 CV risk factor (current smoking, SBP > 140 mmHg, LDL >  2.5 mmol/L, TC/HDL-ratio > 8, chronic renal impairment (age <  65 years: eGFR < 60 ml/min/1,73 m2; age ≥ 65 years: eGFR < 45 ml/min/1,73 m2, and/or (micro)albuminuria).
- The CV risk of the patient is managed in primary care, not in the hospital or outpatient clinic by a medical specialist
- Age between 40 and 80 years

**Table 6:** Risk enhancing factors [7]

	Not risk enhancing	Mildly risk enhancing	Strongly risk enhancing*
First-degree relative with CVD	No	1 family member < 65 years	> 2 family members with CVD < 65 years or 1 < 60 years
Physical activity	≥ 30 min/d, ≥ 5 d/wk	< 30 min/d, ≤ 5 d/wk	Sedentary
Body mass index	BMI < 30 kg/m2	BMI 30–35 kg/m2	BMI > 35 kg/m2
eGFR	< 65 years: > 60 ml/ min/1,73 m2	< 65 years: 30–60 ml/ min/1,73 m2	All ages: < 30 ml/ min/1,73 m2
	≥ 65 years: > 45 ml/ min/1,73 m2	≥ 65 years: 30–45 ml/ min/1,73 m2	

*CVD* = Cardiovascular disease; *eGFR* = estimated glomerular filtration rate; *d* = day or days; *wk*. = week. *In patients with rheumatoid arthritis a high disease activity is a strongly risk enhancing factor

Exclusion criteria for all patients:

- Diabetes mellitus, as these patients are already included in a disease management programme for diabetes mellitus
- Limited life expectancy, as assessed by the GP
- Cognitive impairment, as assessed by the GP
- No Dutch language proficiency
- Staying abroad for longer than 3 months during the duration of the study.
- The CV risk of the patient is managed in the hospital or outpatient clinic by a medical specialist

### Recruitment of patients for the ZWOT-CASE study

The source population consists of 56 general practices. All practices were invited to participate in the study. Eventually, 26 general practices agreed to participate (17 in the intervention group and 9 in the usual care group).

#### Intervention group

Between September and December 2016 general practices randomly invited eligible patients for an intake visit for the integrated CVRM programme. After 1 yr of follow-up, these patients are invited for the study until enough patients are included. The invitation for the study will be sent just before the yearly follow-up visit in the CVRM programme. This visit will be used as the endpoint visit for the study. Just before this follow-up visit, the patients receive a letter from their GP to inform them about the study. If the patient agrees to participate, informed consent is obtained during the follow-up visit.

Patients will be selected in such way, that 50% will be below 65 years and 50% over 65 years, to achieve a reasonable distribution across age categories.

#### Usual care group

In order to create the same study population as in the intervention group, we identify patients in the usual care group according to the protocol (‘Organized Practice’) as described before, including systematically screening of the practice population based on ICPC-coded diagnoses and ATC-codes, and manually checking of medical records. As the general practices in the usual care group do not start with the integrated care programme for CVRM, patients in the usual care group will not be invited for an intake consultation at baseline. Subsequently, a risk profile based on complete data on age, sex, smoking status, blood pressure and lipid spectrum may be missing. In that case, patients can be included if the 10-yeas risk is at least 10%, based on the data that are available. For example, a 55-year old male patient with a missing smoking status and missing total cholesterol/ HDL- cholesterol ratio can be classified as having a CV risk > 10% based on a known systolic blood pressure of 160 mmHg.

Patients in the usual care group are matched to patients in the intervention group. Therefore, the patient in the usual care group will only be invited for the study after the patient in the intervention group agreed to participate. Patients in the usual care group will be invited by letter and subsequently by telephone. If this patient does not agree with participation in the study, the second matched patient from the usual care group will receive an invitation. If the second matched patient also does not agree with participation, we will randomly invite one of the remaining patients from the usual care group who were not invited for the study.

#### Matching

Patients in the usual care group are consecutively matched with the intervention group according to age (per 5 years age categories), gender and risk group (high CV risk or CVD) at the beginning of the study. Each patient in the intervention group is matched to 2 patients in the usual care group. These patients in the usual care group are randomly selected from the eligible population in practices delivering usual care.

### Study procedures

Patients will be identified at the beginning of the study in order to be able to analyse factors such as mortality and comorbidity during follow-up. The study starts when patients in the intervention group visit the general practice for an intake consultation (t = 0). After the intake visit the 1 yr follow-up period commences. After 1 yr of follow-up, patients are invited for an endpoint visit. A questionnaire will be attached to the invitation letter.

**Fig. 1:**
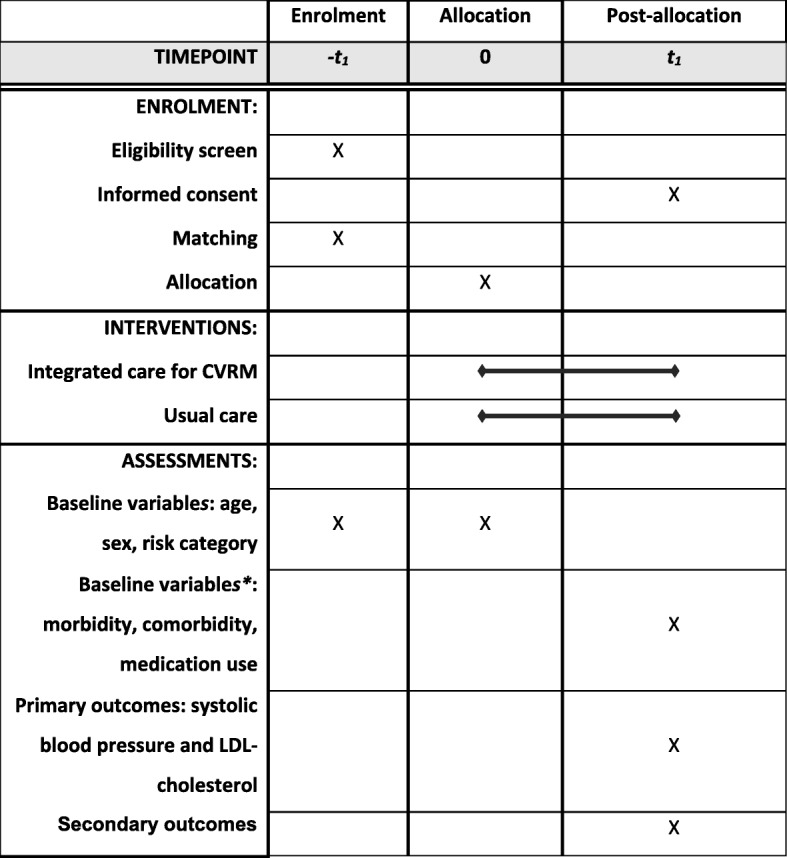
Schedule of enrolment, interventions, and assessments. *These baseline variables are collected retrospectively after one year of follow-up

Prior to the endpoint visit, all patients who agree with participation in the study will be asked to fill out the questionnaire at home. The timeline of the study procedures is represented in Fig. 1.

#### Ethical aspects and informed consent

General practices in both groups will not be informed about which patients are identified at baseline to prevent any influence on their management. Consequently, it will not always be possible to take into account the life expectancy, cognitive function and language skills of the identified patients at baseline, as information on these exclusion criteria is not always adequately registered in the medical records. Just before the end of follow-up, GPs will be informed about the identified patients and asked to assess these exclusion criteria.

Also, at baseline patients in both groups will not yet be informed about the study to prevent that they are aware of being observed and modify their behaviour (Hawthorne effect). Based on the Dutch law for data protection, obtaining informed consent for the identification of patients is not necessary. All obtained data (age, gender and risk category) during the identification will be processed pseudo anonymised and the key to the data will be kept within the general practices. The researchers do have access to this information. Baseline data will be collected retrospectively for all patients as patients are not invited for a baseline visit in the context of the study.

Written informed consent is obtained by the GP or PN during the end-point visit and includes an agreement stating that he or she i) is sufficiently informed, had the opportunity to ask additional questions and had enough time to make a decision; ii) agrees with voluntary participation and at any time can withdraw from participation; iii) agrees with use of medical data and data of questionnaires for the purposes described in the information form; iv) agrees with the storage of the study data for 15 years after this study.

The study was reviewed by the Isala hospital Review Board and exempted from full assessment under the Medical Research Involving Human Subjects Act on the 16th of June 2016 (reference number 16.06104).

### Outcomes and data collection

An overview of the outcomes of the ZWOT-CASE study is shown in Table 7. The primary outcome is systolic blood pressure and LDL-cholesterol. Patients fill out a questionnaire prior to the endpoint-visit including physical activity (squash questionnaire), quality of life (EQ-5D and SF-12), employment (iPCQ), patient satisfaction regarding the provided care (Patient Reported Experience Measure (PREM)), self-management (Patient Activity Measure (PAM)), and anxiety and depression (Hospital Anxiety and Depression Scale (HADS)). Besides, social status, education (UCC-1 questionnaire) [25], food habits, and CV risk perception are measured by a questionnaire.

**Table 7:** Primary and secondary outcomes

Primary endpoints	
1. Systolic blood pressure	
2. LDL-cholesterol	
Secondary endpoints	
1. 10-years cardiovascular morbidity or mortality risk (percentage) (Risk chart Dutch guideline or SMART)	
2. Smoking status	
3. Body mass index (BMI)	
4. Lifestyle (modification) (smoking cessation, healthy food habits, physical activity, motivation for modification and awareness of received advices with respect to weight, food habits and physical activity in the past year)	
5. Awareness of CVD and cardiovascular risk factors	
6. Use of adequate medication (blood pressure lowering drugs, anticoagulants and lipid lowering drugs)	
7. Morbidity (newly developed CVD)	
8. Developed comorbidity (CVD, diabetes mellitus, COPD, heart failure, atrial fibrillation)	
9. Mortality	
10. Primary treating practitioner (GP or medical specialist)	
11. Health care consumption in the past year	
12. Self-management in the past year (patient knowledge, skills, and confidence in managing one’s health and healthcare) (Patient Activity Measure (PAM))	
13. Self-measurements of blood pressure in the past year	
14. Patient satisfaction regarding the provided care in the past year Patient Reported Experience Measure (PREM)	
15. Quality of life (EQ-5D and SF-12)	
16. Anxiety and depression (Hospital Anxiety and Depression Scale (HADS)	
17. Cost-efficiency (iPCQ)	

* HIS = general practice information system

Further, a blood sample is taken for measurement of lipids (total cholesterol, HDL-cholesterol, TC/HDL-cholesterol ratio, LDL-cholesterol, triglycerides), renal function (creatinine, MDRD), glucose and for patients with CVD hs-CRP.

The endpoint visit consists of the same components as the intake consultation in the integrated programme for CVRM as described before. Data collected during the endpoint visit will be registered in the electronic medical record.

An up-to-date 10-years cardiovascular morbidity or mortality risk will be estimated according to the algorithm of the Dutch national guideline for CVRM. This algorithm is based on the SCORE-function, adapted to the Dutch population and converted from a mortality risk to morbidity and mortality risk (based on the MORGEN-cohort and the Rotterdam Study-cohort) [26–29]. The risk chart takes into account age, sex, smoking status, systolic blood pressure and total cholesterol-HDL cholesterol ratio [23, 24]. For patients taking blood pressure or lipid lowering drugs, the actual SBP and cholesterol levels during treatment are used. For patients with CVD the SMART-function will be used to calculate the CV risk [30]. This function is based on age, sex, smoking status, systolic blood pressure, history of diabetes mellitus, ischemic heart disease, cerebrovascular disease, aortic aneurysm, peripheral arterial disease, time since first diagnosis of CVD, HDL-cholesterol, total cholesterol, renal function (eGFR), and high-sensitivity CRP.

Furthermore, baseline data and data concerning health care use in the past year and in the period prior to the study will be collected by scrutinizing the electronic medical files.

Finally, GPs will be asked to complete a survey about the CVRM care, including questions on the practice setting (rural/urban, solo/group), organization of their practice, availability of trained PNs, CVRM programme and possibilities to refer for lifestyle treatment (social map).

### Sample size calculation

Calculation of the sample size is based on a reduction in SBP and LDL-cholesterol in the intervention group after 1 year of follow-up. We consider a 5 mmHg absolute reduction in SBP and a 0.3 mmol/L reduction in LDL-cholesterol in the intervention group as clinically relevant [5, 31]. We assume that SBP and LDL-cholesterol levels in the usual care group remain stable. To detect these differences we need a sample size of 370 patients in the intervention group. This calculation is based on an alpha of 0.05, a power of 80%, and an intra-cluster correlation coefficient of 0.05 for the general practice cluster level. Furthermore, we take into account the response rate. We expect that the response rate in the intervention group will be 70%. This results in a sample size of 587 patients in the intervention group. The intervention patients are matched to patients from the usual care group. In the usual care group we estimated a lower response rate of 50%, as these patients might be less used to visit the general practice compared to the probably regularly controlled patients in the intervention group. Therefore, each intervention patient will be matched with 2 patients from the usual care group. This results in 587 × 2 = 1174 patients in the usual care group. The intervention group and usual care group are both divided into two groups (patients with CVD and patients with high CV risk) equal in size. The intervention group is selected from 17 general practices and the usual care group from 9 general practices.

### Statistical analyses

The aim of the main analysis is to compare the SBP and LDL-cholesterol levels after 1 yr of follow-up between patients in the intervention group receiving integrated care for CVRM and the patients in the usual care group receiving usual CVRM care. For the main analysis we will use linear regression. For secondary outcomes, linear regression will be used for continuous outcomes, logistic regression for dichotomous outcomes and multinomial logistic regression for categorical outcomes. All analyses will be corrected for clustering.

Given that patients of clusters of patients are not randomly allocated to the intervention or usual care group, we anticipate that differences in baseline risk might be present between both groups. Hence, we will adjust the analyses for these confounders, including patient characteristics and practice characteristics. To do such adjusted analysis we a priori define the following baseline covariates which are well-known to be related to the outcomes: i) patient characteristics, including relevant medication use, such as blood pressure lowering drugs [32], anticoagulants [33] and lipid lowering drugs [34], relevant comorbidity, including COPD [35], heart failure [36, 37], atrial fibrillation [38], renal failure [39], ii) practice characteristics, such as involved disciplines in CVRM care (including number of PNs) [31], number of patients, and number of GPs. Results will be reported both without (i.e. crude results) and after correction for confounders. Confounders are defined a priori and not selected based on statistical significance.

Furthermore, we will examine whether the effect of the integrated care for CVRM is modified by differences in the following practice characteristics: practice organization (solo/duo/group), availability of CVRM protocol and existence of other disease management programmes (COPD, DM). This will be done by adding interaction effects.

All analyses are applicable to patient data matched on age and gender.

## Discussion

Disease management programmes for CVRM are gradually implemented in Western countries. So far it is unclear whether such integrated programmes have a positive effect on cardiovascular risk factors and this may lead to discussions between GPs, health insurers and policy makers. Previous studies are heterogeneous in studied interventions and study populations differ substantially in for example included risk categories and the way they are selected, e.g. by active screening or not. Also, adequate comparisons with control groups are lacking [20].

In the region of Zwolle, we have the opportunity to compare integrated care for CVRM with usual care. The aim of the ZWOT-CASE study is to evaluate the effectiveness of integrated care for CVRM compared to usual care in real practice. Since this is a pragmatic study, some choices in the study design were made that may have some methodological drawbacks. Below the major strengths and limitations of our study will be discussed.

### Strengths

First, the pragmatic design of the ZWOT-CASE study will give insight into whether integrated care for CVRM compared to usual care is effective in a real world environment. If integrated care for CVRM has a positive effect on our outcomes, it supports the idea that the intervention is beneficial in daily practice.

Furthermore, all the general practices are affiliated to the care group ‘Medrie’. The regional implementation of integrated care for CVRM will lead to a more uniform intervention and to less differences between general practices in the intervention group and consequently to less between-cluster variability. Also, the care group reached an agreement on integrated care for CVRM with the health care insurance for the coming 3 yrs, ensuring an adequately financed and stable health care environment during the follow-up of the ZWOT-CASE study. GPs in the usual care group are also members of the care group. This makes the usual care group probably more comparable with the intervention group with regard to socioeconomic characteristics and available opportunities for referrals.

Finally, a strength of the ZWOT-CASE study is the lenient inclusion criteria, i.e. patients with a CV risk of > 10%. Since usually more strict inclusion criteria are used in other regions, subgroup analysis will enable us to translate our results to other regions.

### Limitations

One limitation is the lack of random allocation to the two arms. This might lead to differences in practice characteristics between the intervention group and the usual group, and lead to either an over- or underestimation of the effect of integrated care for CVRM, when these differences are not adequately adjusted for. Besides, there may be differences in given care before implementation of integrated care for CVRM between different practices within the intervention group. This could also influence the effect of integrated care of CVRM. However, due to the complete registration of patient data in the Dutch general practice information system, we will be able to accurately collect patient data on years prior to the study and take into account the given care prior to the intervention. Furthermore, ample measures have been taken (notably matching of patients, multivariable analyses) to prevent confounding in our study.

Since patients will be selected from different general practices, we have to deal with a cluster effect. The intervention under study may be heterogeneous [40]. For example, differences in practice size, practice facilities and space, training of GP and staff, availability of supportive staff, time-management, attitude towards prevention and the GP-patient relationship might lead to cluster-level differences.

Another limitation of our study design is that it is not possible to blind GPs, PNs and participants for the intervention. Blinding is impossible due to the nature of the intervention. To minimize bias and maximize the validity of the results, participants are selected just before the end of the study. Also, during follow-up the general practices are not informed about which patients are identified as eligible to participate in the study.

A disadvantage of the regional approach of our study is the risk of contamination between the usual care group and the intervention group. Usual care may change in the direction of the intervention and therefore the effect of the intervention may be underestimated. However, as GPs in the usual care group do not use the guideline for the implementation of integrated care for CVRM, do not register patient data in an information system for integrated care, and yearly benchmark meetings are not mandatory, we expect the effect of contamination to be minimal. We will collect data on provided CVRM care to assess contamination.

The regional approach of this study could reduce the generalizability of the findings to other regions in the Netherlands. However, we expect that most of the integrated care programmes for CVRM are based on the same Dutch guideline for CVRM and the same international guidelines for CVRM.

The follow-up of 1 year could be too short to analyse the full effect of integrated care for CVRM. However, we expect that the largest gained improvements in cardiovascular risk factors will occur within 1 yr. Therefore, a follow-up of 1 yr will be sufficient to assess the effectiveness of the intervention on these cardiovascular risk factors. But, if integrated care for CVRM is effective, it would be interesting to analyse whether the effect continues. Also, improvements in cardiovascular risk factors will only translate in a lower cardiovascular morbidity and mortality in the longer term [3]. To observe the durability of an effect, and to assess the effect of integrated care for CVRM on absolute cardiovascular morbidity and mortality, studies with a longer follow-up period should be conducted.

Finally, a general limitation of studies in general practice is that due to a lot of variation in the organization of primary care across Europe and beyond [41, 42], the results of a study in one country will probably only be generalizable to countries with similarly organised primary health care systems. However, to support the implementation as well as continuation of disease management programmes scientific evidence is needed. Integrated programmes for CVRM have been introduced in Western countries in recent years. Evidence on the effect of such integrated CVRM care is very limited. The ZWOT-CASE study will give insight into the effectiveness of integrated care for CVRM compared to usual care in general practice.

## Data Availability

Not applicable.

## Abbreviations

ATC-codes: Anatomical Therapeutic Chemical-codes
BMI: Body mass index
CCM: Chronic Care Model
CV risk: Cardiovascular risk
CVD: Cardiovascular disease
CVRM: Cardiovascular risk management
DM: Diabetes mellitus
eGFR: Estimated glomerular filtration rate
GP: General practitioner
HDL: High-density lipoprotein
ICPC: International Classification of Primary Care
LDL: Low-density lipoprotein
PN: Practice nurse
SBP: Systolic blood pressure
TC: Total cholesterol
TIA: Transient ischaemic attack
